# Authors' reply

**DOI:** 10.4103/0253-7613.59932

**Published:** 2009-12

**Authors:** Kavitha S. Nair, Kirti V. Patel, Tejal R. Gandhi

**Affiliations:** Department of Pharmacology, Anand Pharmacy College, Opp. Town Hall, Anand-388001, Gujarat, India E-mail: kavitha21_82@yahoo.co.in

Sir,

It appears from the above discussion that the modulatory role of cytochromes on pioglitazone is doubtful, but numerous evidences has accumulated after the above-cited references that have supported the role of pioglitazone as cytochrome p450 enzyme inducer (although weak).[1,2]

The benefits of ANOVA and post hoc Tuckey can be considered to be equivalent to Chi-square test.
